# Regulatory mechanisms of two epiphytic cultivation modes of *Cunninghamia lanceolata* on growth, disease resistance and root-stem characteristics of *Dendrobium devonianum*

**DOI:** 10.1186/s12870-026-09227-w

**Published:** 2026-06-11

**Authors:** Chunmei Sun, Qingli Han, Xiahong He, Hoang Van Sam, Wen Fu, Junrong Tang, Na Zhang

**Affiliations:** 1 Yunnan Key Laboratory of Conservation and Utilization of Under Forest Resources, College of Forestry, Southwest Forestry University, Kunming, 650224 China; 2 Vietnam National University of Forestry, Hanoi, 13417 Vietnam; 3 Yunnan Provincial Plant Protection and Plant Inspection Station, Kunming, 650034 China

**Keywords:** *Dendrobium devonianum*, Understory cultivation, Epiphytic cultivation mode, Root endophytic microbes, Stem metabolome, Disease resistance

## Abstract

*Dendrobium devonianum* (purple-skinned *Dendrobium*) is a pillar crop in Longling, Yunnan, the “Hometown of Purple-skinned Dendrobium”. Understory wild-simulated epiphytic cultivation is critical for high-quality organic production and regional industrial sustainability. This study compared two *Cunninghamia lanceolata-*based modes (dead tree frame cultivation vs. living standing tree cultivation) to optimize *D. devonianum* cultivation. Results showed that: (1) dead tree frame increased stem diameter (+ 28.0%), stem length (+ 21.6%), and fresh weight (+ 8.4%, *P* < 0.05) but caused significantly higher rust incidence (68.40% ± 0.05% vs. 41.80% ± 0.08%) and black spot disease index (14.60 ± 2.94 vs. 7.88 ± 1.12, *P* < 0.05). (2) High-throughput sequencing revealed the living standing tree group had significantly higher abundances of Actinobacteria (+ 35.2%), Acidobacteriota (+ 42.1%), and Bacteroidota (+ 28.6%, *P* < 0.05), which were negatively correlated with disease incidence. In contrast, the dead tree frame group was dominated by opportunistic pathogen-related phyla. (3) LC-MS/MS analysis showed that dead tree frame cultivation upregulated stress-responsive metabolites (fatty acyls, prenol lipids), while living standing tree cultivation significantly enriched flavonoids (+ 36.7%) and polysaccharides (+ 169.8%, *P* < 0.05). The living standing tree mode achieves synergistic improvements in disease resistance and medicinal quality, supporting a regulatory cascade linking cultivation mode, root endophytic microbes, metabolic reprogramming, and plant performance. This makes it more suitable for high-quality, eco-friendly *D. devonianum* cultivation in Longling, providing technical support for industrial upgrading.

**Graphical abstract:**

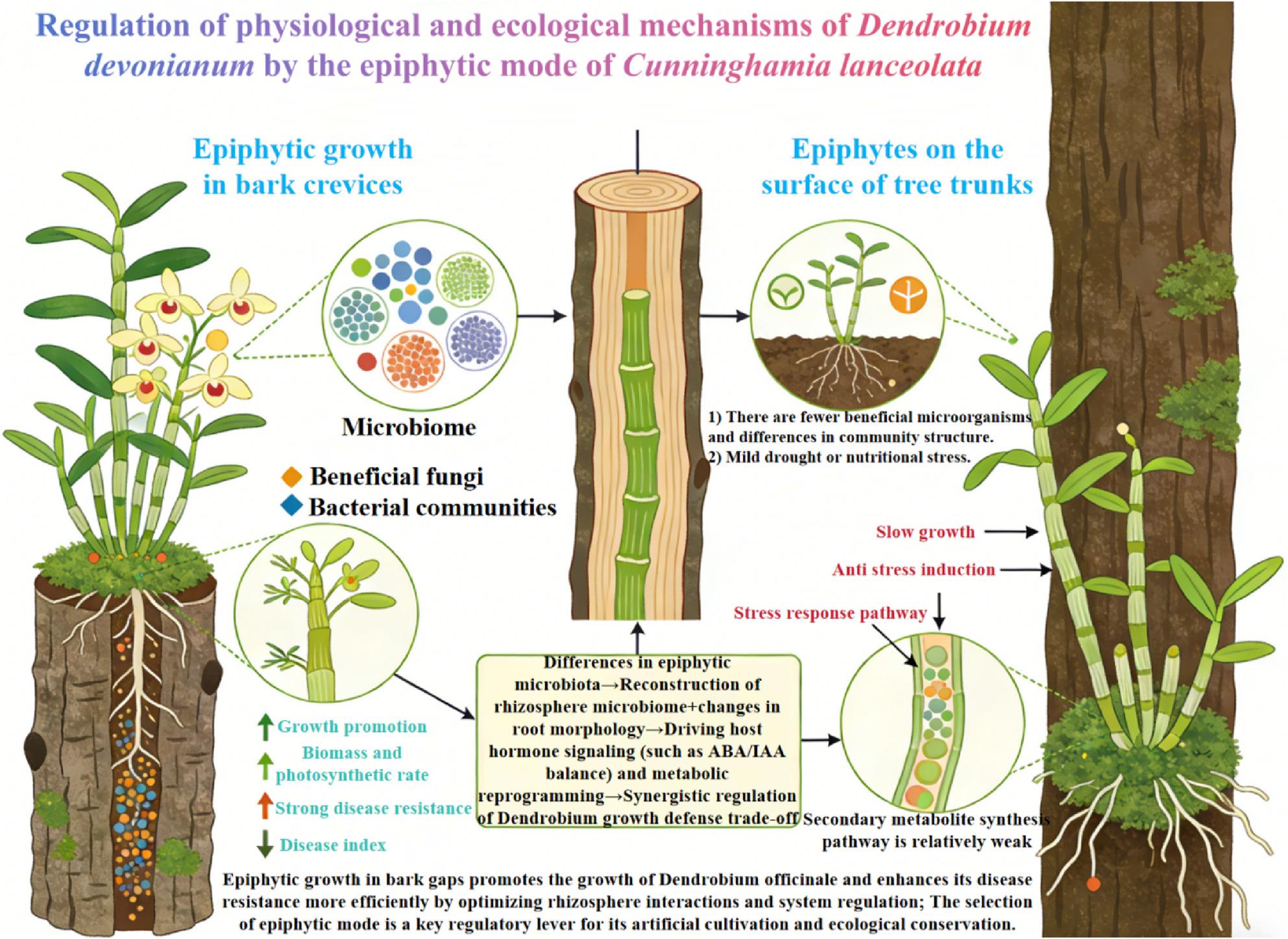

## Introduction

*Dendrobium devonianum*, also known as purple-skinned Dendrobium, is an epiphytic herbaceous plant of the genus *Dendrobium* in the Orchidaceae family [1]. Its stems are rich in bioactive compounds such as polysaccharides, bibenzyls, and alkaloids, endowing it with significant medicinal and economic value. Longling county is recognized as the “hometown of purple-skinned Dendrobium”, and the Dendrobium industry has become a core pillar for consolidating poverty alleviation achievements and promoting rural revitalization [2]. However, due to habitat destruction and over-harvesting, wild *D. devonianum* resources are increasingly depleted, and the species has been listed as a nationally protected wild plant in China. Consequently, wild-simulated understory epiphytic cultivation has emerged as an essential approach to balance resource conservation with industrial sustainable development [3].

*Cunninghamia lanceolata* is locally abundant and is the most commonly used substrate for epiphytic Dendrobium cultivation in Longling County, Making it an appropriate choice for comparing different epiphytic modes [4].

Cultivation mode is a key factor regulating agronomic traits, disease resistance, and medicinal quality of *D. devonianum*. Previous studies have shown that different cultivation modes can reshape the root endophytic microbial community and further influence plant metabolite accumulation [5]. However, most existing studies have focused on single or pairwise associations within the “cultivation mode → root endophytic microbes → metabolite” cascade. Few studies have integrated microbial and metabolic changes with overall plant performance, including agronomic traits, disease resistance, and medicinal quality, into a unified systemic framework. Notably, disease resistance directly affects yield stability, while agronomic traits and medicinal quality are the two core output indicators of Dendrobium cultivation. Therefore, the complete regulatory cascade of “cultivation mode → root endophytic microbes → metabolic reprogramming → agronomic traits, disease resistance, and medicinal quality” and its underlying synergistic mechanisms remain largely unclear. How the functional links between microorganisms and host metabolism ultimately shape plant integrated performance has become a theoretical bottleneck restricting the high-quality development of the Dendrobium industry.

To address this knowledge gap, this study focuses on understory epiphytic *D. devonianum* and compares two typical epiphytic cultivation modes using *Cunninghamia lanceolata*: living standing tree cultivation (hereafter referred to as living standing tree) and dead tree frame cultivation using withered tree logs (hereafter referred to as dead tree frame). *C. lanceolata* was selected as the substrate because it is locally abundant, widely used by farmers, and represents the conventional local practice for epiphytic Dendrobium cultivation in Longling [5]. By integrating root endophytic microbial community and metabolomic analyses, we systematically elucidate the synergistic regulatory mechanisms by which different cultivation modes affect the agronomic traits, disease resistance, and medicinal quality of *D. devonianum*. The findings aim to provide a scientific basis for optimizing high-quality and efficient cultivation techniques and to offer theoretical support for the green and sustainable development of the purple-skinned Dendrobium industry in Longling.

## Materials and methods

### Plant materials and experimental setup

The experiment was conducted at an understory *Dendrobium devonianum* cultivation base in Longling County, Yunnan, China (24.597978°N, 98.711975°E). The site lies in a coniferous and broad-leaved mixed forest dominated by *Cunninghamia lanceolata*, *Cinnamomum camphora*, and *Celtis kunmingensis*. Longling County has a mean annual temperature of 16.4 °C, annual precipitation of 1699.2 mm, 84% average relative humidity, and 73.62% forest coverage. The experimental site is at 1400–1700 m elevation, with an annual average temperature of approximately 17 °C. Overstory canopy closure is 0.6–0.7, and understory light intensity ranges from 8000 to 15,000 lx, with no direct solar radiation. The test material, *Dendrobium devonianum*, was propagated via tissue culture [6]. The plant material was obtained with formal written consent from the Dendrobium Germplasm Resource Conservation and Research Center. A voucher specimen (accession No. Dd-SWFU-2023-041) has been deposited in the laboratory of the Yunnan Key Laboratory of Conservation and Utilization of Under Forest Resources, Southwest Forestry University, and is available upon request. After rooting, the seedlings were transplanted into an understory nursery substrate for one year of acclimatization. At the time of transplanting for the experiment, the seedlings were 5–8 cm in height, with a stem diameter of 0.15–0.20 cm, and were robust and free from pests and diseases. The growth substrate consisted of *Cunninghamia lanceolata* bark mixed with a small amount of moss. No chemical fertilizers or synthetic pesticides were applied. During the dry season, irrigation was supplied via a spray system every 2–3 days, with no additional fertilization. Two cultivation modes were implemented: (1) Dead Tree Frame Cultivation using *C. lanceolata*: Felled *C. lanceolata* logs were constructed into horizontal triangular prism-shaped dead tree frame. Seedlings were fixed onto the frames using plastic tying ropes. (2) Living Standing Tree Cultivation using *C. lanceolata*: Seedlings were directly fixed onto living *C. lanceolata* trees using plastic tying ropes. Both modes were established within the same forest stand, ensuring consistent environmental conditions. Cultivation, Sampling and Sample Preparation: Seedlings were transplanted in April 2023. Whole fresh plant samples from both cultivation modes were collected in July 2024. Samples were immediately frozen in liquid nitrogen, transported to the laboratory, and stored at -80 °C for subsequent analysis. Roots were used for microbial diversity analysis, and stems were used for metabolite profiling. Three biological replicates were established for each cultivation mode, with each replicate consisting of a pooled sample from 10 individual plants (Table 1).

**Table 1:** Sample numbers of *D. devonianum* under two different cultivation models of *C.lanceolata*

Dendrobium varieties	Cultivation mode	Sample ID	Dendrobium age/month
*D. devonianum*	Dead Tree Frame Epiphytic	root(GDZ), stem (DZ)	15
	Living Standing Tree Epiphytic	root(GDS), stem (DS)	15

GDZ and DZ are root and stem samples from the Dead Tree Frame Epiphytic mode, respectively; GDS and DS are root and stem samples from the Living Standing Tree Epiphytic mode, respectively

### Agronomic traits and disease assessment

Three biological replicates were established per cultivation mode (living standing tree vs. dead tree frame). For each cultivation mode (living standing tree vs. dead tree frame), three plots (replicates) were randomly arranged within the forest. Within each plot, 100 plants were assessed (five sampling points × 20 trees/frames per point, one plant randomly taken per tree/frame). The 100 measurements were averaged to obtain one value per plot, resulting in a statistical sample size of *n* = 3 per mode. Independent t-tests were used to compare the two modes. Disease incidence and index were calculated based on a 5-grade scale. The incidence and disease index of black spot and rust were recorded and calculated. Disease severity was rated using a whole-plant 5-grade scale according to the method reported by Liu et al. [7]. The following formulas were used for disease calculation:

$$\begin{aligned} &Disease\,incidence(\%)\\&=(Number\,of\,infected\,plants / \\& \quad Total\,number\,of\,plants\,investigated)\\& \quad \times100\% \end{aligned}$$

$$\begin{aligned} Disease\,index=&\left[100\times\sum\left(Number\,of\,plants\,in\,each\,grade\times\,Grade\,value\right)\right]\\&/(Total\,number\,of\,plants\,investigated \\&\times\,Value\,of\,the\,highest\,grade ) \end{aligned}$$

Statistical analysis was performed using SPSS 26.0 software. Normality was assessed using the Shapiro-Wilk test, and homogeneity of variances was assessed using Levene’s test. As all data met the assumptions of normality and homogeneity of variance, differences between the two cultivation modes were compared using an independent samples t-test, with a significance level set at *P* < 0.05.

### Microbial community profiling

Endophytic microbial DNA was extracted using the OMEGA Soil DNA Kit. Dendrobium roots were rinsed with sterile water to remove soil residues, surface-sterilized with 75% ethanol (3 min) and 2.5% NaClO (5 min), and rinsed 5 times with sterile water. The final rinse was plated on culture medium to confirm sterilization (no colony growth). The bacterial 16S rRNA gene (V3-V4 region) and the fungal ITS region were amplified with primer pairs 338F/806R and ITS1F/ITS2R, respectively. Amplicons were sequenced on an Illumina MiSeq platform. Paired-end reads were denoised using DADA2 in QIIME 2 (2019.4) with forward and reverse reads truncated to 240 bp and 200 bp, respectively, maxEE = 2.0 for both, and no 5’ trimming. Rarefaction was performed at 95% of the lowest-depth sample (40,000 reads for bacteria, 80,000 for fungi), and rarefaction curves (10 depth steps, 10 iterations) confirmed sufficient coverage. Taxonomy was assigned using the classify-sklearn algorithm with a 0.7 confidence threshold against Greengenes (13.8) for bacterial 16 S rRNA and UNITE (8.0) for fungal ITS sequences. Beta-diversity was assessed using weighted UniFrac distances, and PERMANOVA (999 permutations) tested for significant differences between cultivation modes.

### Metabolite profiling and analysis

#### Metabolite extraction

Lyophilized stem samples were ground and extracted with pre‑chilled methanol‑acetonitrile‑water (2:2:1, v/v/v) using ice‑bath sonication. After being placed at ‑20 °C for 1 h, the extracts were centrifuged at 14,000 g for 20 min at 4 °C. The supernatant was vacuum‑dried, reconstituted in cold methanol‑acetonitrile (1:1, v/v), sonicated, incubated at ‑20 °C, and centrifuged. The final supernatant was dried, dissolved in 50% acetonitrile, and filtered through a 0.22‑µm cellulose acetate membrane prior to LC‑MS/MS analysis.

#### LC‑MS/MS detection and data processing

Metabolite profiling was performed using LC‑MS/MS. Raw data were processed with MS‑DIAL software. According to the Metabolomics Standards Initiative (MSI) guidelines, metabolites were annotated at MSI Level 2 (putative annotation), based on matching accurate mass (< 10 ppm error) and MS/MS spectra (< 0.01 Da error) against the HMDB, MassBank, and BioZerone‑DB standard libraries. Differential metabolites were screened by combining multivariate and univariate statistical analyses: variable importance in projection (VIP) > 1.0 from the partial least squares-discriminant analysis (PLS-DA) model, and *P* < 0.05 from independent samples t-test. Statistical analyses were performed using SPSS 26.0 and GraphPad Prism 9. The permutation test (*n* = 200) was performed to assess model overfitting. The intercept values were R² = 0.3324 and Q² = − 0.55. Since the Q² intercept was well below the commonly accepted threshold of 0.05, the established PLS-DA model was confirmed to be robust and without overfitting.

### Determination of nutritional components

In July 2024, samples were collected from each tree from four cardinal directions, with two clusters randomly selected per direction. From each cluster, three mature stems were collected, and the two clusters were combined to form one sample. Within each block, eight cluster samples were further pooled, resulting in three block replicates per treatment. The samples were immediately frozen in liquid nitrogen and stored at − 80 °C. The detection methods were as follows: cellulose content was determined using the sulfuric acid-anthrone colorimetric method [8]; total alkaloids were analyzed using the Reinecke salt colorimetric method [9]; total flavonoids were determined by spectrophotometry [10]; and total polysaccharides were measured according to the national standard GB/T 15,672 − 2009 [11]. Each sample was measured in triplicate.

## Results and analysis

### Differences in agronomic traits and disease incidence

Statistical analysis revealed significant differences (*P* < 0.05) in agronomic traits and disease incidence of *D.devonianum* between the two cultivation modes (Table 2). Under the dead tree frame cultivation mode, the plant exhibited significantly superior agronomic performance, including greater stem diameter (0.32 ± 0.05 cm), stem length (11.99 ± 1.92 cm), fresh weight (5.18 ± 0.04 g), and plant height (10.54 ± 2.38 cm), compared to the living standing tree mode. The increment of stem diameter, stem length, and fresh weight in the dead tree frame mode was 28.0%, 21.6%, and 8.4%, respectively, with extremely significant statistical differences (*P* < 0.01), indicating that the dead tree frame mode has a strong promoting effect on the biomass accumulation of *D. devonianum* [12].

**Table 2:** Agronomic traits and major disease occurrence of *D. devonianum* under two cultivation modes using *C. lanceolata* (mean ± SD)

Measurement	Living Standing Tree Epiphytic Cultivation	Dead Tree Frame Epiphytic Cultivation	*P*-value
Stem Diameter (cm)	0.25 ± 0.02b	0.32 ± 0.05a	0.002
Stem Length (cm)	9.86 ± 1.83b	11.99 ± 1.92a	0.006
Plant Height (cm)	5.76 ± 1.39b	10.54 ± 2.38a	< 0.001
Fresh Weight (g)	4.78 ± 0.12b	5.18 ± 0.04a	< 0.001
Rust Incidence (%)	41.80 ± 0.08b	68.40 ± 0.05a	< 0.001
Rust Disease Index	15.56 ± 5.28b	32.24 ± 3.85a	< 0.001
Black Spot Incidence (%)	27.00 ± 0.04b	39.40 ± 0.04a	< 0.001
Black Spot Disease Index	7.88 ± 1.12b	14.60 ± 2.94a	< 0.001

Values represent mean ± SD. Within each row, different superscript letters indicate a significant difference between cultivation modes. *P* < 0.05 indicates a significant difference, *p* < 0.01 indicates an extremely significant difference. Values of *p* < 0.001 are reported as < 0.001

However, disease incidence was also significantly higher in the dead tree frame cultivation (Table 2). The incidence of rust reached 68.40% ± 0.05%, representing a 63.6% increase over the living standing tree mode (41.80% ± 0.08%), while the rust disease index (32.24 ± 3.85) was 107.2% higher. Similarly, the incidence and disease index of black spot were significantly elevated under dead tree frame cultivation compared to the living standing tree mode [13]. Correlation analysis between agronomic traits and disease indices showed that stem diameter and fresh weight were significantly positively correlated with rust incidence (*r* = 0.73 and 0.68, *P* < 0.05), suggesting that the rapid growth of *D. devonianum* in the dead tree frame mode may be accompanied by enhanced susceptibility to pathogens [14].

### Analysis of root endophytic microbial community

#### Summary of high-throughput sequencing results

**Fig. 1:**
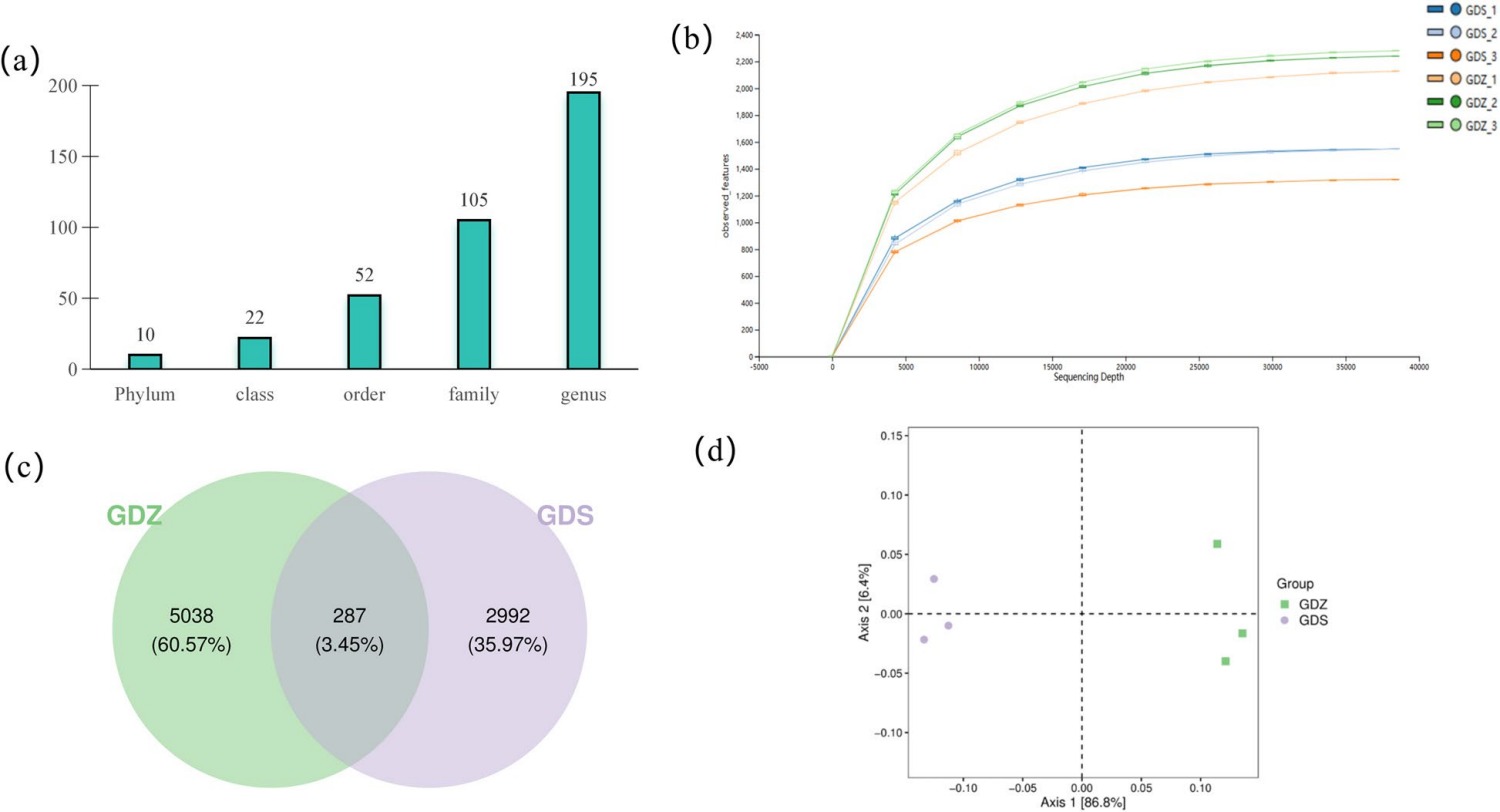
Statistics of high-throughput sequencing results for bacterial community diversity in *D. devonianum* roots. Note: (**a**) Taxonomic composition at phylum, class, order, family, and genus levels; (**b**) Rarefaction curves reflecting sequencing depth sufficiency; (**c**) Venn diagram showing shared and unique ASVs between groups; (**d**) PCoA of the bacterial community in under two cultivation Modes

Sequencing of the 16 S rRNA gene yielded 544,929 high‑quality reads after quality control, which were clustered into 384 amplicon sequence variants (ASVs). These ASVs spanned 10 phyla, 22 classes, 52 orders, 105 families, and 195 genera (Fig. 1a). Rarefaction curves indicated that the sequencing depth was sufficient to capture the majority of bacterial diversity (Fig. 1b). Venn diagram analysis (Fig. 1c) revealed that 287 ASVs were shared between the GDZ (dead tree frame cultivation) and GDS (living standing tree cultivation) groups [15]. The GDZ group contained 5,038 unique ASVs (60.57%), while the GDS group contained 2,992 unique ASVs (35.97%). The number of unique bacterial ASVs in the dead tree frame group was 1.68 times that of the living standing tree group, indicating that the bacterial community richness of *D. devonianum* roots in the dead tree frame mode was significantly higher, but the proportion of unique ASVs was lower [16]. The number of bacterial ASVs in the roots of plants under living standing tree cultivation was significantly lower than that under dead tree frame cultivation [17], indicating a substantial difference in bacterial community structure between the two cultivation modes (Fig. 1d).

**Fig. 2:**
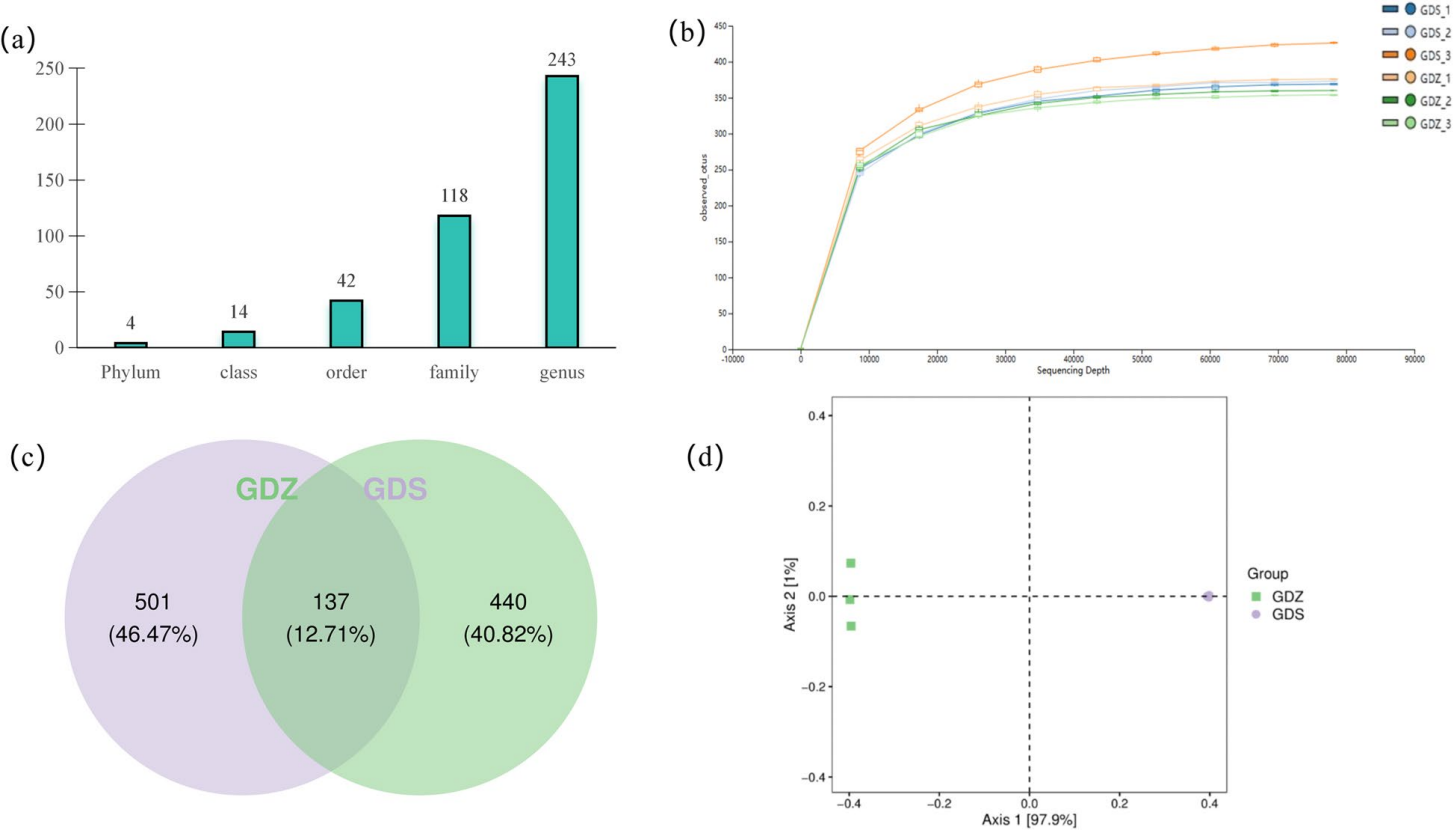
Statistics of high-throughput sequencing results for fungal community diversity in *D. devonianum* roots. Note: (**a**) Taxonomic composition at phylum, class, order, family, and genus levels; (**b**) Rarefaction curves reflecting sequencing depth sufficiency; (**c**) Venn diagram showing shared and unique ASVs between groups; (**d**) PCoA plot displaying fungal community separation between cultivation modes

Sequencing of the ITS region yielded 586,119 high-quality reads, with 467,360 sequences retained after quality control. These were clustered into 431 ASVs, spanning 4 phyla, 14 classes, 42 orders, 118 families, and 243 genera (Fig. 2a). Rarefaction curves reached a plateau, indicating sufficient sequencing depth to capture fungal diversity (Fig. 2b). Venn diagram analysis revealed 137 shared ASVs between the GDZ and GDS groups, with 501 (46.47%) and 440 (40.82%) unique ASVs, respectively (Fig. 2c). Principal coordinate analysis (PCoA) further demonstrated distinct fungal community structures between the two cultivation groups (Fig. 2d), with the first two principal components explaining 79.3% of the total variation, indicating that cultivation mode is a key factor driving the differentiation of fungal community structure [18].

#### Alpha and beta diversity analysis

Alpha diversity analysis showed significant differences in the Shannon and Simpson indices of the root endophytic bacterial communities between the two cultivation modes (*P* < 0.05). For endophytic fungal communities, the Chao1 index did not differ significantly (*P* = 0.243), while the Shannon index differed significantly (*P* = 0.007). The richness (Chao1 index: 1478.603 ± 131.48) and diversity (Shannon index: 8.290 ± 0.14) of root endophytic bacterial communities in plants under living standing tree cultivation (GDS) were significantly lower than those under dead tree frame cultivation (GDZ; Chao1: 2228.233 ± 75.37, Shannon: 9.459 ± 0.09). The bacterial Chao1 index of the dead tree frame group was 1.51 times that of the living standing tree group, suggesting that the endophytic bacterial community richness in the root zone of *D. devonianum* under the dead tree frame mode is significantly higher, which may be related to the more complex organic matter composition of the withered wood substrate [19]. In contrast, endophytic fungal community richness (Chao1: 391.320 ± 33.48) was slightly higher under living standing tree cultivation than under dead tree frame cultivation (Chao1: 363.366 ± 11.39), but its diversity was significantly lower (Table 3).

**Table 3:** Alpha diversity indices of endophytic bacteria and fungi in the roots of *D. devonianum* under different cultivation modes (Mean ± SD)

Group	Endophytic bacteria			Endophytic fungi		
	Chao1	Shannon	Simpson	Chao1	Shannon	Simpson
GDS	1478.603 ± 131.48	8.290 ± 0.14	0.989	391.320 ± 33.48	4.295 ± 0.08	0.882
GDZ	2228.233 ± 75.37	9.459 ± 0.09	0.996	363.366 ± 11.39	4.696 ± 0.03	0.907
*t*-value	-8.568	-12.379	-13.069	1.369	-7.973	-12.565
*p*-value	0.001	< 0.001	< 0.001	0.243	0.007	< 0.001

*p* < 0.05 indicates a significant difference, *p* < 0.01 indicates an extremely significant difference. Values of *p* < 0.001 are reported as < 0.001

**Fig. 3:**
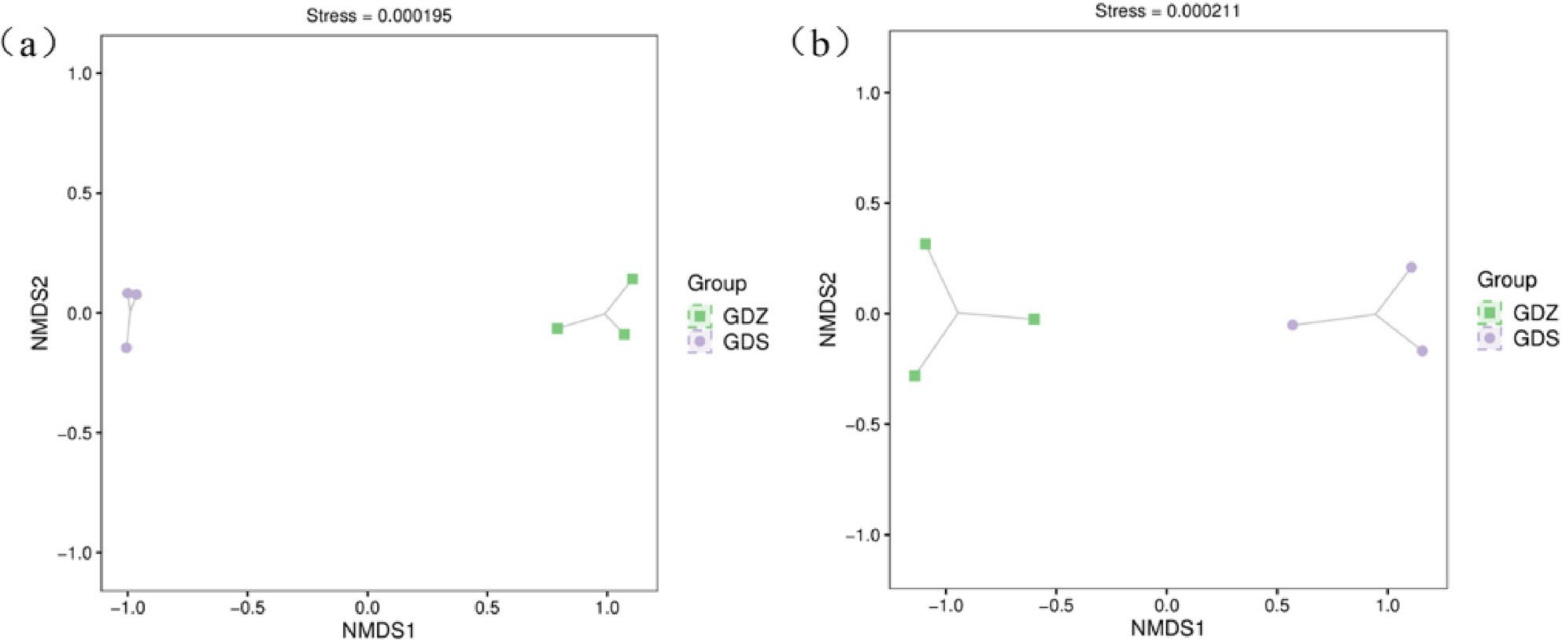
NMDS analysis of bacterial and fungal endophytic communities in the roots of *D. devonianum* under different cultivation modes. Note: (**a**) Bacterial endophytic community NMDS plot (Stress = 0.000195); (**b**) Fungal endophytic community NMDS plot (Stress = 0.000211). Different colors represent different cultivation modes, reflecting clear community separation

Beta diversity analysis based on NMDS (Fig. 3) showed Stress values of 0.000195 for bacteria and 0.000211 for fungi (both < 0.2), indicating reliable ordination results and demonstrating significant differences in the root endophytic community structure between the two cultivation modes [20]. Non-parametric permutation tests (Adonis) confirmed that the differences in bacterial and fungal community structures between the two modes were statistically significant (*P* = 0.001 and *P* = 0.002, respectively), further verifying that cultivation mode is a key driver of microbial community differentiation [21].

#### Compositional differences at phylum and genus levels

**Fig. 4:**
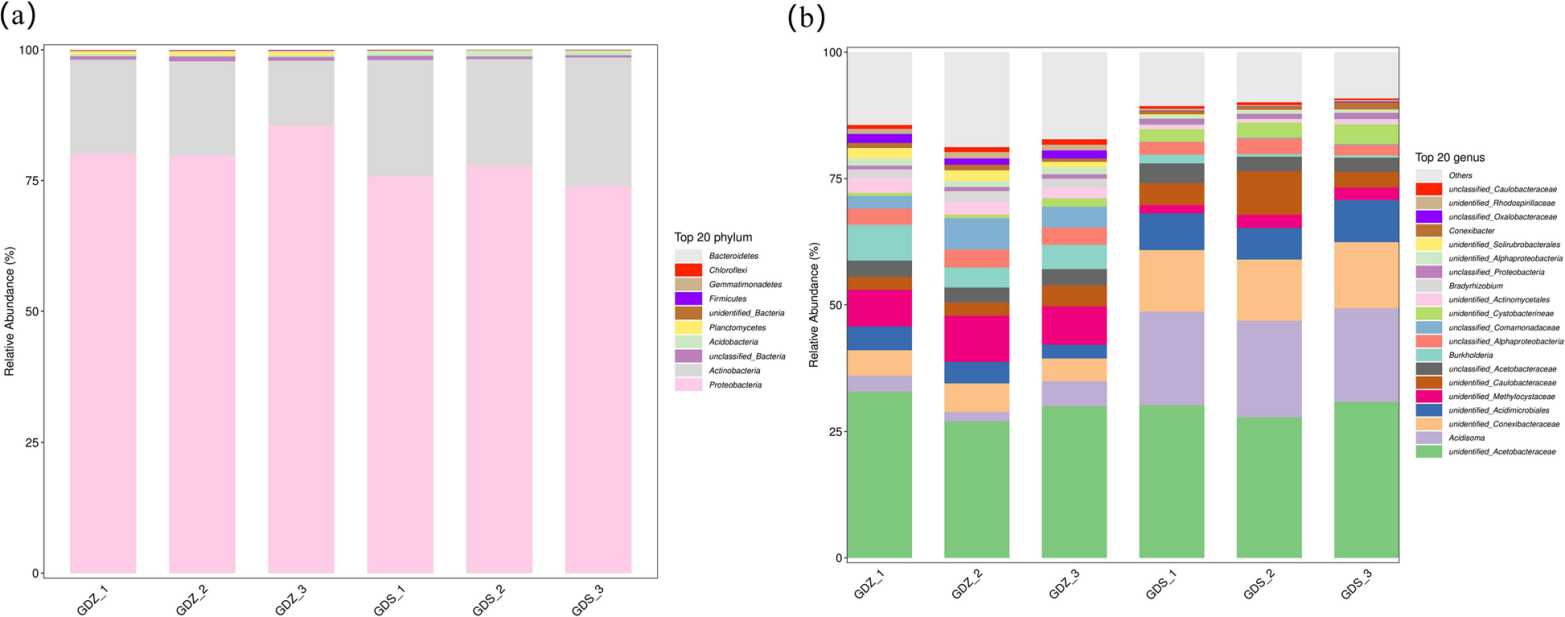
Taxonomic composition of the root endophytic bacterial community in *D. devonianum* at the phylum and genus levels under different cultivation modes. Note: (**a**) Phylum-level composition; (**b**) Genus-level composition, showing dominant taxa and their relative abundances in each mode

Analysis of 16 S rRNA sequencing data identified a total of 10 bacterial phyla in the roots of *D. devonianum* under both cultivation modes, including Proteobacteria, Actinobacteria, unclassified_Bacteria, and Acidobacteria, among others—with Proteobacteria being the dominant phylum across all samples (Table 3; Fig. 4). Differential abundance analysis revealed that the dead tree frame cultivation group exhibited significantly higher relative abundances of Proteobacteria, Planctomycetota, and unclassified_Bacteria (*P* < 0.05), while the living standing tree cultivation group showed significantly higher abundances of Actinobacteria, Acidobacteriota, and Bacteroidota (*P* < 0.05) [22].

Correlation analysis (Pearson correlation) between endophytic microbial relative abundance data and disease incidence data showed that the relative abundances of Actinobacteria, Acidobacteriota, and Bacteroidota were significantly negatively correlated with the incidence of rust and black spot [23] (*r* range: -0.75 to -0.82, *P* < 0.05). Among them, Acidobacteriota had the strongest negative correlation with rust incidence (*r* = -0.82, *P* < 0.01), followed by Actinobacteria (*r* = -0.80, *P* < 0.01).

At the genus level, dominant genera shared between the two modes included unidentified_Acetobacteraceae, *Acidisoma*, unidentified_Conexibacteraceae, and unidentified_Acidimicrobiales. *Acidisoma* (belonging to Acidobacteriota) and unidentified_Conexibacteraceae (belonging to Actinobacteria) were significantly more abundant in the living standing tree group [24] (*P* < 0.05). Additionally, the relative abundances of *Acidisoma* and unidentified_Conexibacteraceae were significantly higher in the living standing tree group, while the dead tree frame group showed higher abundances of unidentified_Acetobacteraceae, *Burkholderia*, and *Bradyrhizobium* (*P* < 0.05).

#### Fungal community composition at phylum and genus levels

**Fig. 5:**
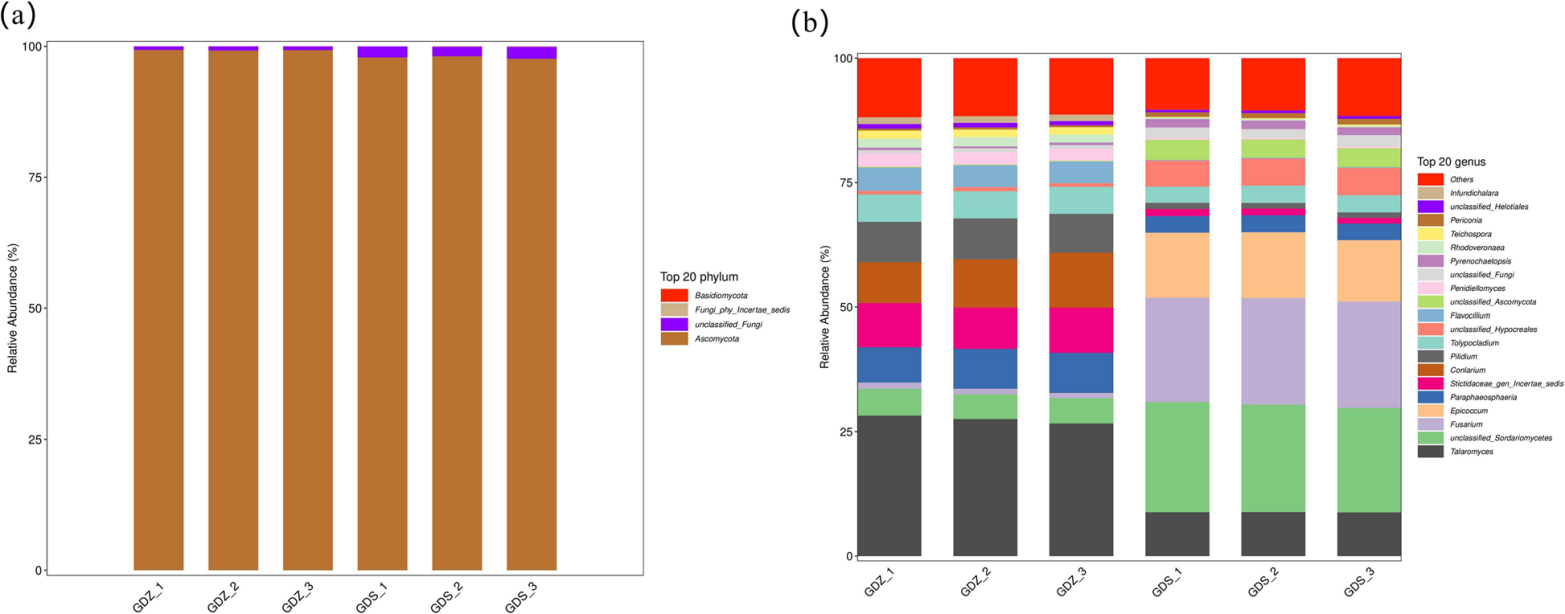
Taxonomic composition of the root endophytic fungal community in *D. devonianum* at the phylum and genus levels under different cultivation modes. Note: (**a**) Phylum-level composition; (**b**) Genus-level composition, showing dominant taxa and their relative abundances in each mode

At the phylum level, ITS sequencing identified four fungal phyla: Ascomycota (dominant in all samples), unclassified fungi, Fungi_phy_Incertae_sedis, and Basidiomycota, with no significant difference in the relative abundance of Ascomycota between the two cultivation modes [25]. At the genus level, shared dominant genera included *unclassified_Sordariomycetes*, *Fusarium*, *Epicoccum*, *Talaromyces*, and *Paraphaeosphaeria* [26]. The living standing tree cultivation group showed significantly higher abundances of *unclassified_Sordariomycetes*, *Fusarium*, and *Epicoccum*, whereas the dead tree frame cultivation group exhibited significantly higher abundances of *Talaromyces*, *Paraphaeosphaeria*, and other genera, including *Sticicdaceae-gen-incertae-sedis*, *Conlarium*, *Pilidium*, and *Tolypocladium* (*P* < 0.05) (Fig. 5).

The relative abundance of *Fusarium* (a genus with potential pathogenic members) in the living standing tree cultivation group was 1.2 times that of the dead tree frame cultivation group, while the incidence of rust and black spot in the living standing tree cultivation group was significantly lower than in the dead tree frame cultivation group (*P* < 0.05). Combined with alpha diversity analysis, which indicated a significantly higher fungal Shannon index in the dead tree frame cultivation group (*P* < 0.05), these results demonstrate distinct fungal community composition and structure between the two cultivation modes, with richer fungal diversity observed under dead tree frame cultivation [27].

### Differences in stem metabolite composition

#### Principal component analysis (PCA)

**Fig. 6:**
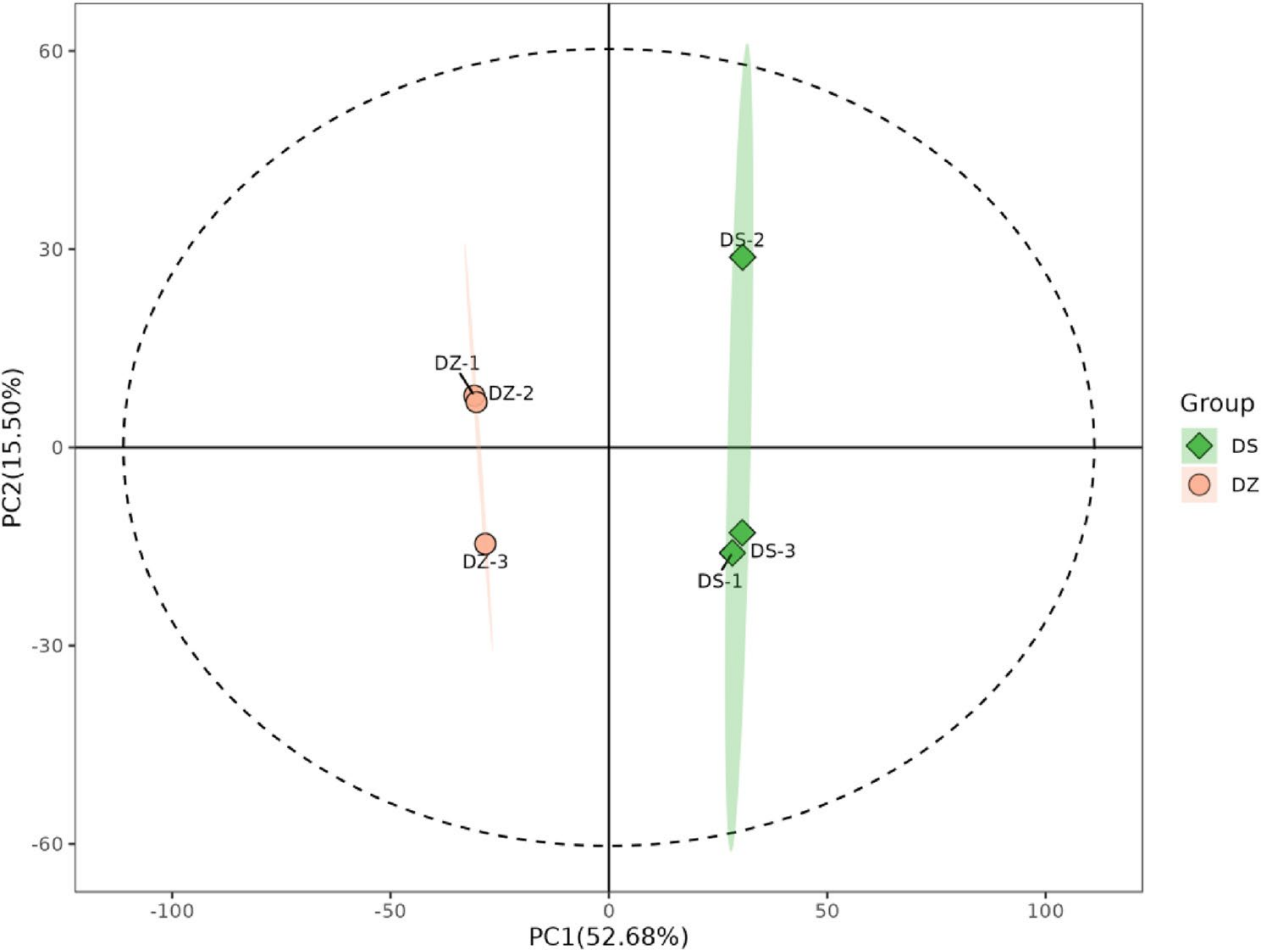
PCA Score Plot of Overall Samples from *D. devonianum* in Positive and Negative Ion Modes under Different Cultivation Modes. Note: PC1 explains 52.68% of total variance, PC2 explains 15.50%; different colors represent different cultivation modes, showing clear sample clustering and separation

PCA results (Fig. 6) showed that the first and second principal components (PC1 and PC2) explained 52.68% and 15.50% of the total variance, respectively. All samples fell within the 95% confidence intervals, and samples from the two cultivation modes displayed clear separation along PC1 [28]. The samples of the two cultivation modes were clustered into two distinct groups without overlap, suggesting that the epiphytic mode has a significant regulatory effect on the metabolic profile of *D. devonianum* stems, and the metabolic differences between the two modes are stable and reliable [29]. This indicates that the epiphytic mode significantly influenced the synthesis and accumulation of metabolites in *D. devonianum* stems.

#### Screening of differential metabolites

Based on the predefined screening criteria (VIP > 1.0, *P* < 0.05), a total of 892 significantly differential metabolites were identified between the two cultivation modes, of which 555 were up-regulated and 337 were down-regulated in the dead tree frame group relative to the living standing tree group. The number of up-regulated metabolites was 1.65 times that of down-regulated ones, suggesting that the dead tree frame mode mainly promotes metabolite accumulation, while the living standing tree mode inhibits part of metabolite synthesis and favors the enrichment of specific compounds [30].

**Fig. 7:**
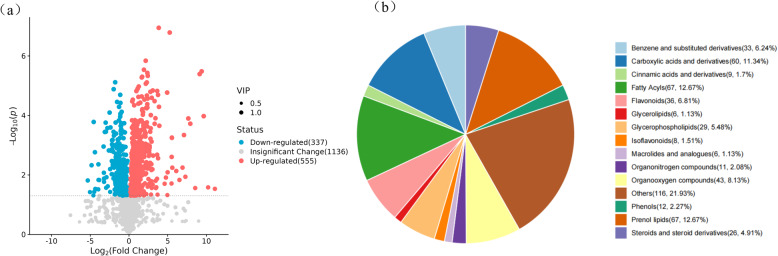
Screening results of significant differential metabolites in the stems of *D. devonianum* under different cultivation modes. Note: (**a**) Volcano plot of DZ vs. DS (x-axis: log2(Fold Change), y-axis: -log10(*P*-value)); (**b**) Pie chart of metabolite classification, showing the proportion of each metabolite class among differential metabolites

The major categories of differential metabolites included fatty acyls, prenol lipids, organooxygen compounds, carboxylic acids and derivatives, and flavonoids—these five classes accounted for 78.3% of all differential metabolites, representing the core metabolic pathways regulated by cultivation modes [31]. Notably, β-ionone (fatty acyls), decursin, and naringenin (flavonoids) were significantly up-regulated in the dead tree frame group (Fig. 7).

#### Hierarchical clustering analysis of differential metabolites

**Fig. 8:**
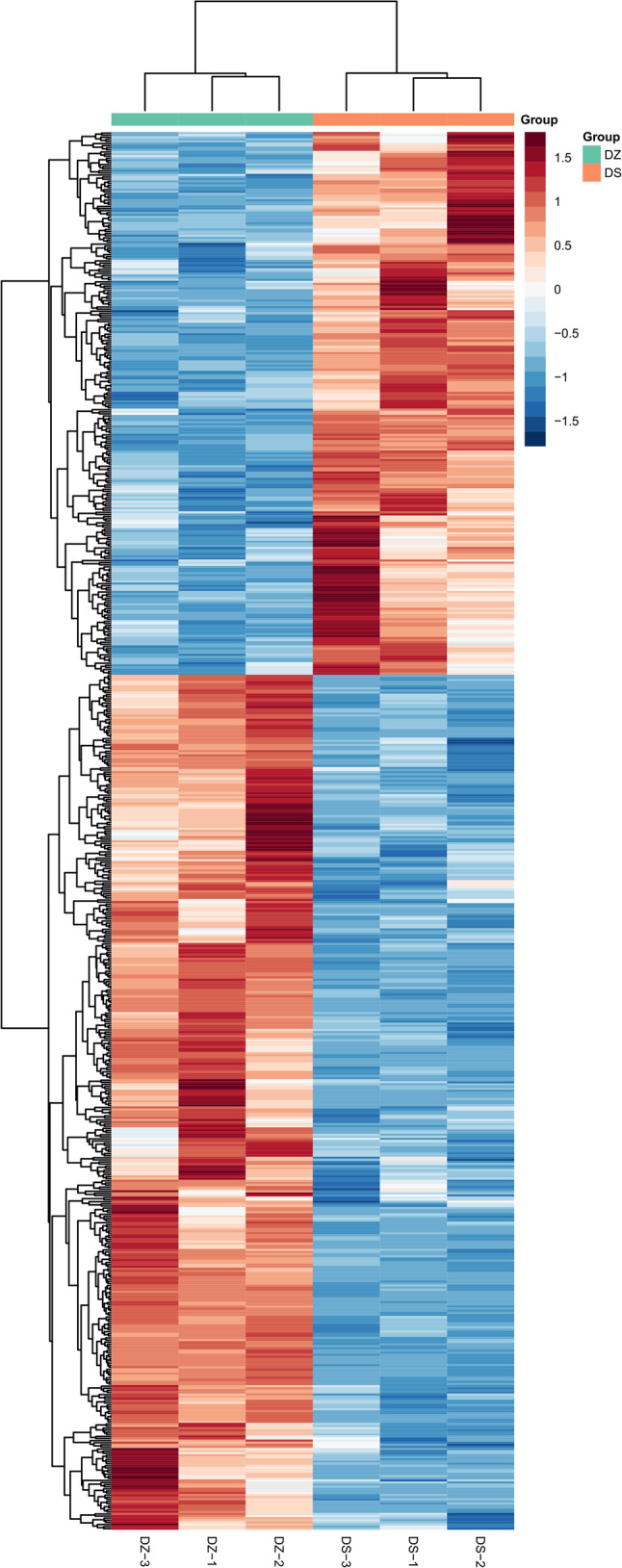
Clustered heatmap of differentially accumulated metabolites in the stems of *D. devonianum* under different cultivation modes. Note: Rows represent differential metabolites, columns represent samples; red indicates up-regulated metabolites, blue indicates down-regulated metabolites, showing clear clustering of samples from the same cultivation mode

Hierarchical clustering of the top 50 differential metabolites clearly separated the two cultivation modes into distinct clusters (Fig. 8), confirming highly significant and stable metabolic differences between groups (consistent with the 892 differential metabolites identified in Sect. Screening of differential metabolites).

Classification statistics (Table 4) revealed distinct metabolic preferences between cultivation modes: the dead tree frame group had higher abundances of seven metabolite classes, with fatty acyls (50 Up-regulated, 17 Down-regulated) and prenol lipids (47 Up-regulated, 20 Down-regulated) accounting for 38.2% of up-regulated metabolites.These are typical stress-responsive metabolites, suggesting that *D. devonianum* in this mode may be under continuous stress. Additionally benzene and derivatives (21 Up-regulated, 12 Down-regulated), organooxygen compounds (22 Up-regulated, 21 Down-regulated), steroids and derivatives (21 Up-regulated, 5 Down-regulated), phenolic compounds (9 Up-regulated, 3 Down-regulated), and organonitrogen compounds (10 Up-regulated, 1 Down-regulated), while the living standing tree group showed higher abundances of flavonoids (15 Up-regulated, 21 Down-regulated), glycerophospholipids (13 Up-regulated, 16 Down-regulated), and cinnamic acids and derivatives (3 Up-regulated, 6 Down-regulated), and carboxylic acids and derivatives were relatively balanced between the two modes (30 Up-regulated, 30 Down-regulated).

**Table 4:** Statistics of significantly differential metabolites in *D. devonianum* between dead tree frame and living standing tree cultivation using *C. lanceolata*

Number	Class of Metabolites	Total Number of Differential Metabolites	Up-regulated	Down-regulated
1	Fatty Acyls	67	50	17
2	Prenol lipids	67	47	20
3	Carboxylic acids and derivatives	60	30	30
4	Organooxygen compounds	43	22	21
5	Flavonoids	36	15	21
6	Benzene and substituted derivatives	33	21	12
7	Glycerophospholipids	29	13	16
8	Steroids and steroid derivatives	26	21	5
9	Phenols	12	9	3
10	Organonitrogen compounds	11	10	1
11	Cinnamic acids and derivatives	9	3	6

Data are based on screening results of significantly differential metabolites (VIP > 1.0 and *P* < 0.05). Up‑regulation/down‑regulation are defined relative to the living standing tree cultivation group as the baseline. Up‑regulation in the dead tree frame group indicates significantly higher metabolite content compared to the living standing tree group; down‑regulation indicates significantly lower content

#### Differences in nutrient accumulation

The detection results (Table 5) indicated that the contents of cellulose and alkaloids in *D. devonianum* under dead tree frame cultivation were significantly higher than those under living standing tree cultivation *(P* < 0.05). In contrast, the contents of flavonoids and polysaccharides in the living standing tree group were significantly higher than those in the dead tree frame group (*P* < 0.05): specifically, polysaccharide content increased by 169.8% and flavonoid content by 36.7% compared to the dead tree frame group [32]. Correlation analysis between differential metabolites and nutrient components showed that the up-regulated flavonoid metabolites in the living standing tree group (e.g., naringenin, quercetin) were significantly positively correlated with total flavonoid content (*r* = 0.81, *P* < 0.01), and the accumulation of polysaccharide-related metabolites (e.g., glucose polymers) was consistent with the increase in total polysaccharide content in Table 5.

**Table 5:** Accumulation of nutritional components in the stems of *D. devonianum* under different cultivation modes

Indicator (mg/g)	Dead Tree Frame Epiphytic	Living Standing Tree Epiphytic
Cellulose	32.9 ± 1.88a	20.3 ± 0.379b
Total Flavonoids	2.43 ± 0.153b	3.67 ± 0.058a
Total Alkaloids	7.61 ± 0.422a	6.93 ± 0.084b
Total Polysaccharides	22.9 ± 0.513b	61.7 ± 1.45a

All values are mean ± SD (*n* = 3), different superscript letters (a, b) in the same row indicate significant difference at *P* < 0.05 (independent samples t-test)

## Discussion

The two epiphytic cultivation modes of *Cunninghamia lanceolata* (living standing tree vs. dead tree frame) produced contrasting effects on *Dendrobium devonianum*. The living standing tree mode enhanced disease resistance but reduced biomass, while the dead tree frame mode promoted growth but increased disease susceptibility. This growth-defense trade-off is discussed below in relation to root endophytic microbial community structure and metabolomic profiles.

The following interpretations are based on correlational evidence and should be considered speculative rather than definitive. Direct experimental validation (e.g., microenvironmental monitoring, strain inoculation assays, co-occurrence network analysis, functional assays) is needed to confirm the proposed mechanisms.

### Balancing agronomic traits and disease resistance: A critical criterion for selecting optimal cultivation modes

Balancing agronomic traits and disease resistance is a critical criterion for selecting optimal *D. devonianum* cultivation modes, as this balance directly shapes the sustainability and economic benefits of understory cultivation systems.

The present results demonstrated that dead tree frame cultivation significantly improved agronomic traits but increased disease susceptibility compared with living standing tree cultivation (e.g., stem diameter + 28.0%; rust incidence + 63.6%). This observation aligns with the findings of Wang et al. [32] and Liu et al. [7], suggesting a growth-defense trade-off in epiphytic orchid cultivation.

From a practical perspective, for high-quality organic production with reduced pesticide use, living standing tree cultivation appears more suitable, as it achieves better disease resistance and medicinal quality. The underlying mechanisms of this trade-off remain to be elucidated.

### Root endophytic microbes community: Core internal factor regulating disease resistance

The root endophytic microbial community may act as a key factor linking cultivation modes to *D. devonianum* disease resistance [33]. High-throughput sequencing results revealed distinct root endophytic microbial community structures between the two cultivation modes: the living standing tree group exhibited significantly higher abundances of Actinobacteria, Acidobacteriota, and Bacteroidota (relative abundances increased by 35.2%, 42.1%, and 28.6%, respectively).

The associations between these microbial taxa and disease resistance are supported by three lines of correlational evidence, summarized in Table 6. First, correlation analysis showed that the relative abundances of Actinobacteria, Acidobacteriota, and Bacteroidota were strongly negatively correlated with rust and black spot incidence (*r* = -0.75 to -0.82, *P* < 0.05). Second, at the genus level, *Acidisoma* (Acidobacteriota) and *unidentified_Conexibacteraceae* (Actinobacteria) were significantly enriched in the living standing tree group; these genera are documented to produce antifungal compounds. Third, independent studies have reported that Actinobacteria produce antibiotics, while Acidobacteriota and Bacteroidota compete for nutrients and secrete antibacterial metabolites, representing classic biocontrol mechanisms [34].

**Table 6:** Summary of evidence supporting the association between root endophytic microbes and disease resistance of *D. devonianum*

Evidence Type	Data/Evidence Source	Core Basis	Logic Chain
Correlation analysis (measured data)	This study Sect. Differences in agronomic traits and disease incidence (disease incidence) + Sect. Compositional differences at phylum and genus levels (microbial abundance), Pearson correlation analysis	Actinobacteria/Acidobacteriota/Bacteroidota abundances were strongly negatively correlated with rust/black spot incidence (*r* = -0.75~ -0.82, *P* < 0.05), with Acidobacteriota showing the strongest correlation (*r*=-0.82)	Microbial abundance ↑ → Disease incidence ↓ → Direct association between microbes and disease resistance
Genus-level functional characteristics (measured data + literature)	This study Sect. Compositional differences at phylum and genus levels (genus-level abundance)	*Acidisoma* (Acidobacteriota) and *unidentified_Conexibacteraceae* (*Actinobacteria*) were significantly enriched in the living standing tree group; these genera are documented to produce antifungal compounds	Enrichment of functional genera → Enhanced pathogen suppression → Indirect confirmation of microbial biocontrol effects

All measured data are from this study; literature support corresponds to the reference numbers in the text, ensuring traceability and reliability

Collectively, these lines of evidence suggest that the enrichment of these microbial taxa in the living standing tree group may be associated with disease suppression. Consistent with recent reports on natural bioactive compounds for sustainable crop disease control [35], the enrichment of beneficial microbial taxa in the living standing tree group may contribute to disease suppression, which may collectively help explain the lower disease incidence observed in this group. However, it is important to note that these associations are correlational. Targeted strain isolation and functional assays (e.g., in vitro antagonism tests, plant inoculation experiments) are needed to confirm causality.

Although the relative abundance of *Fusarium* was higher in the living standing tree group than in the dead tree frame group, the living standing tree group showed lower disease incidence. This pattern may be explained by differences in fungal community composition between the two modes. The dead tree frame group had significantly higher fungal diversity (Shannon index: 4.696 vs. 4.295, *P* < 0.05) and higher abundances of saprotrophic genera (e.g., Talaromyces, Paraphaeosphaeria), which may competitively reduce the relative abundance of *Fusarium* [36–38]. In contrast, the living standing tree group had lower fungal diversity and fewer saprotrophic fungi, resulting in a higher relative abundance of *Fusarium*. However, whether the *Fusarium* strains in the living standing tree group are pathogenic to *D. devonianum* remains unknown, as strain-level identification and functional assays were not performed. It is known that many *Fusarium* species can colonize plants asymptomatically as endophytes and only become pathogenic under host stress or ecosystem disturbance. Further studies are needed to determine the pathogenicity of the specific *Fusarium* strains present in each cultivation mode.

### Metabolomic and bioactive component differences between cultivation modes

Metabolomic analysis further uncovered differences in metabolic profiles between the two cultivation modes. Using LC-MS/MS technology, 892 significantly differential metabolites (VIP > 1.0 and *P* < 0.05) were identified.

The dead tree frame group showed higher abundances of stress-responsive metabolites, including fatty acyls (50 types up-regulated) and prenol lipids (47 types up-regulated), which may reflect adaptive responses to disease stress [39]. In contrast, the living standing tree group showed significant enrichment of core medicinal components: flavonoids (relative content increased by 36.7%) and polysaccharides (relative content increased by 169.8%, as shown in Table 5).

The two cultivation modes directed metabolite accumulation toward different pathways: the dead tree frame group toward stress defense, while the living standing tree group toward medicinal quality. The observed differences in metabolic profiles suggest metabolic reprogramming in response to different disease pressures. The dead tree frame group accumulated more stress-responsive metabolites, whereas the living standing tree group accumulated more medicinal components. These patterns are consistent with the higher disease pressure observed in the dead tree frame group [40, 41].

The enrichment of flavonoids and polysaccharides in the living standing tree group is consistent with the medicinal quality orientation of *D. devonianum*, further suggesting that living standing tree cultivation may be more conducive to producing high-quality medicinal Dendrobium [42]. However, the specific molecular mechanisms underlying these metabolic shifts remain to be elucidated, and direct experimental validation (e.g., targeted metabolite profiling or gene expression studies) is needed to confirm these observations.

### Limitations and future perspectives

This study has the following limitations. First, we did not measure microenvironmental parameters (e.g., temperature, relative humidity, light intensity, and bark/substrate nutrient content). Therefore, the observed differences in root endophytic microbial community structure and host metabolism between the two epiphytic modes are correlative, and our interpretations regarding microenvironmental influences remain speculative. Second, we did not perform co-occurrence network analysis to identify keystone microbial taxa, limiting our ability to fully decipher microbial community interactions. Third, no strain-level functional validation (e.g., pathogenicity assays, inoculation experiments) was performed. While Actinobacteria, Acidobacteriota, and Bacteroidota were significantly enriched in the living standing tree group and correlated negatively with disease incidence, their specific roles in disease suppression remain unconfirmed.

Future studies should: (i) incorporate continuous monitoring of microenvironmental factors to establish causal relationships; (ii) employ microbial co-occurrence network analysis to identify keystone taxa driving the assembly of beneficial microbial communities; (iii) conduct targeted isolation of dominant strains (e.g., *Fusarium*, Actinobacteria, Acidobacteriota) and perform functional assays to verify their biological roles; and (iv) evaluate the long-term effects of different cultivation modes on soil ecology and biodiversity [43–45].

Despite these limitations, this study provides a comprehensive multi-omics comparison of two typical epiphytic cultivation modes and offers practical guidance for optimizing high-quality *D. devonianum* production in Longling, Yunnan.

## Conclusions

This study investigated the regulatory mechanisms of two epiphytic cultivation modes of *Cunninghamia lanceolata* (living standing tree vs. dead tree frame) on *Dendrobium devonianum*. The key findings are:

i. Growth-disease resistance trade-off. Dead tree frame cultivation improved agronomic traits but increased disease susceptibility, while living standing tree cultivation showed lower biomass but stronger disease resistance.
ii. Root endophytic microbial community. Living standing tree cultivation significantly enriched Actinobacteria, Acidobacteriota, and Bacteroidota, which were negatively correlated with disease incidence. These associations suggest a link between beneficial microbes and disease suppression, though causality requires functional validation.
iii. Medicinal quality. Living standing tree cultivation enhanced flavonoids (+ 36.7%) and polysaccharides (+ 169.8%), while dead tree frame cultivation upregulated stress-responsive metabolites (fatty acyls, prenol lipids).
iv. Regulatory cascade. This study reveals a correlative regulatory cascade of cultivation mode → root endophytic microbes → metabolic reprogramming → agronomic traits, disease resistance, and medicinal quality.
v. Practical recommendation. Living standing tree cultivation was associated with higher abundances of beneficial microbes, enhanced medicinal components, and stronger disease resistance, making it suitable for high-quality organic production in Longling, Yunnan.

## Data Availability

All data generated or analyzed during this study are included in the manuscript. The raw sequencing data (if applicable) will be deposited in a public repository (e.g., NCBI SRA, Figshare) upon acceptance of the manuscript. For additional data requests, please contact the corresponding author (Qingli Han:281872516@qq.com) .

## Abbreviations

P.R.China: The People’s Republic of China
LC-MS/MS: Liquid Chromatography - Tandem Mass Spectrometry
MS‑DIAL: MS‑DIal And Lipidomics
HMDB: Human Metabolome Database
*D. devonianum*: Dendrobium devonianum
*C. lanceolata*: Cunninghamia lanceolata
NMDS: Non-metric Multidimensional Scaling
PCA: Principal Component Analysis
